# Super long viewing distance light homogeneous emitting three-dimensional display

**DOI:** 10.1038/srep09532

**Published:** 2015-04-01

**Authors:** Hongen Liao

**Affiliations:** 1 Department of Biomedical Engineering, School of Medicine, Tsinghua University, Beijing 100084, China

## Abstract

Three-dimensional (3D) display technology has continuously been attracting public attention with the progress in today's 3D television and mature display technologies. The primary characteristics of conventional glasses-free autostereoscopic displays, such as spatial resolution, image depths, and viewing angle, are often limited due to the use of optical lenses or optical gratings. We present a 3D display using MEMS-scanning-mechanism-based light homogeneous emitting (LHE) approach and demonstrate that the display can directly generate an autostereoscopic 3D image without the need for optical lenses or gratings. The generated 3D image has the advantages of non-aberration and a high-definition spatial resolution, making it the first to exhibit animated 3D images with image depth of six meters. Our LHE 3D display approach can be used to generate a natural flat-panel 3D display with super long viewing distance and alternative real-time image update.

Autostereoscopic 3D displays^1^,^2^,^3^, including direction-multiplexed and diffraction-based approaches using lenticular lenses, micro lens arrays, and optical gratings, do not require special viewing glasses or tracking devices^4^,^5^,^6^,^7^. However, their limitations, such as narrow viewing angle, low image quality, crosstalk, and shallow image depth, prevent their further promotion in a larger area of naked-eye 3D visualization and wider commercial use^8^,^9^. Light field and multi-direction 3D displays have also been developed to address the wide-angle glasses-free 3D display issue^7^. Large-scale autostereoscopic displays using high-resolution projectors and a combination of optical lenses or diffraction gratings have a similar limitation^10^,^11^,^12^. To promote further application in market-oriented products, it is necessary to break through the bottleneck of current 3D display technologies. Holography can be used to achieve the idea 3D effect^13^,^14^,^15^. However, it is not easy to achieve rapid progress for commercial use in a short period due to various factors, such as dynamic display, real-time generation, and electronic signal transfer for TV presence.

Although numerous autostereoscopic 3D displaying techniques have been developed in the past century, few produce a long-viewing-distance 3D image that has a large image depth of several meters and can be perceived by the naked eye^16^. A 3D image with a long viewing distance or large image depth is an important factor for future 3D visualization devices for common use, such as 3D TVs and other telepresence devices.

As described above, an optical lens or diffraction grating is an essential component for building a flat-panel-type 3D autostereoscopic display. To obtain a higher resolution and large image depth of a 3D image, it is necessary to arrange elemental images on a high-resolution and high-pixel-density flat display at corresponding positions to the optical apparatus. However, the resolution and image depth of the 3D autostereoscopic image reproduced by the optical lens or optical grating deteriorates due to aberrations such as spherical aberration or grating diffraction^17^. The aberration and field curvature are so large that the resolution of the 3D image used optical apparatus are far from the ideal results. This is also a common issue of naked-eye 3D display using binocular, multi-view lens, and multi grating methods.

For this research, we propose an approach for displaying 3D images without using high-pixel-density flat display and optical lens arrays or optical gratings, automatically resolving the optical aberrations problems caused by lenses or gratings.

In conventional 3D displays using optical lenses or gratings, images in the image group irradiated using backlight are projected in 3D space via the corresponding optical apparatus (Fig. 1a), so that the 3D image is displayed as a result of accumulated multi-view rays from the corresponding optical apparatus. On the other hand, this structure can be substituted with a combination of micro lenses and a micro elemental images array (Fig. 1b). If rays from a light beam source (such as laser diodes) are modulated in their luminance and the modulated rays are reflected by the biaxial MEMS scanning mirrors (Fig. 1c) such that the rays are projected in the same spatial positions as shown in Fig. 1b, a 3D image is formed in 3D space as the same image shown in Figs. 1a and 1b. If the rays are scanned at a high speed (for example, more than 60 Hz), the naked eye recognizes the intermittent rays as continuous rays due to after images on the retina, as the same 3D image formed by continuously rays projected from the micro projection array shown in Fig. 1b.

## Results

**Figure 2:**
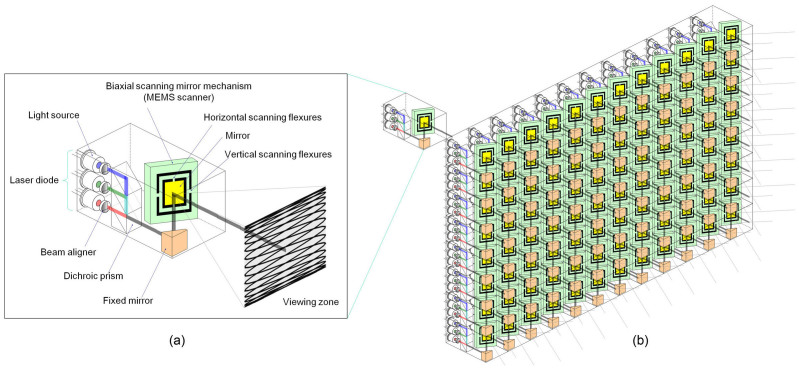
LHE 3D display constructed with MEMS scanning units. (a) Concept of LHE MEMS scanning unit; each unit consists of light source, beam aligners, reflection mirror, and biaxial MEMS scanning mirror. For color image, color beams from diodes are aligned by corresponding beam aligners into intense beams spreading proportional to distance from diodes. Color beams can be collected together into one beam by transmitting through dichroic mirror. Beam is relayed onto biaxial MEMS scanning mirror that reflect beam in Lissajous pattern in space. (b) LHE 3D display constructed by arrangement of MEMS scanning units and corresponding signals input. Each unit corresponds to one pixel shown in 3D display.

To make the proposed approach possible, we present a method for displaying a 3D image by driving multiple LHE MEMS scanning units. Since we use a single-pixel beam to establish a basic unit of an LHE multi-view 3D display, the MEMS scanning unit plays a role of fast projection optics by producing multi-view homogeneous emitting beams with a horizontal and vertical projection angle. Figure 2 illustrates the concept of a MEMS scanning unit designed for the proposed LHE 3D display approach. Each unit consists of a light source, beam aligners, a reflection mirror, and a biaxial MEMS scanning mirror (Fig. 2a). To produce color images, the lights from three laser diodes are combined with a dichroic prism into a single coaxial full-color light beam. The collected beam is reflected by the fixed mirror and transmitted to the biaxial scanning mirror, where the beam is scanned two-dimensionally. The image signals are inputted via the laser diodes in the basic MEMS scanning unit.

The direction of the light rays are controlled using biaxial scanning mechanisms. The MEMS scanning mirror is manufactured as a galvano-mirror comprising a tiny mirror and torsion springs attached to the mirror for support. The mirror can be driven by electro-magnetic force, attraction or repulsion force of static electricity, or piezoelectric force. The single-pixel light beam is scanned to draw a Lissajous figure constituted by sine waves in a horizontal direction and in a vertical direction. The desired 2D scanning can be achieved by properly selecting frequencies and phases of the sine waves in the two directions. The luminance of the laser diode is modulated based on the image signals in accordance with the resonant frequency of the biaxial scanning mirror; thus, 3D images in accordance with the inputted image signals are projected in 3D space.

The MEMS scanning units as elemental pixels are used to construct the LHE 3D display (Fig. 2b). To precisely project the images in the required directions for generating a long-viewing-distance 3D image, the projected directions of each MEMS scanning unit are calibrated before use. We use a computer-assisted geometric correcting approach^12^ in which the image position adjustment parameters are fed into a computer or image processing graphics board and used to adjust each MEMS scanning unit's image projection position (see Method 1, Supplementary Figures 1 and 2) through a serial input. After calibration of the LHE MEMS scanning 3D display, we can input the revised elemental images to each MEMS scanning array so that the impinged light beams are reflected to a predetermined area in space and the 3D image can be spatially formatted.

**Figure 3:**
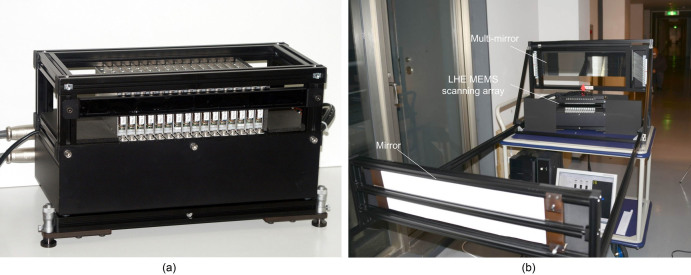
Manufactured LHE 3D display. (a) Manufactured LHE 3D display with 16 MEMS scanning units; (b) LHE 3D display system using MEMS scanning array and mirror array for evaluation of long viewing distance 3D image.

For this study, we fabricated a prototype LHE 3D display for horizontal-parallax-only 3D images and conducted a set of experiments to evaluate the feasibility of the display (Fig. 3a). Multiple MEMS scanning units are aligned horizontally. This display technique makes it easy to create a full-parallax autostereoscopic display by adding vertical arrangement of the MEMS scanning units. Each scanning unit is reconstructed and modified based on a laser projector^18^,^19^ (Microvision SHOWWX + HDMI Laser Projector). The resonant motion of the horizontal scanning flexures and vertical scanning flexures are 18 KHz and 60 Hz, respectively. The frame rate is typically 60 Hz for an 848 × 480 WVGA resolution. We use a neutral density (ND) filter to reduce the brightness so that the 3D image can be viewed by the naked eye. The prototype LHE 3D display was constructed using 16 MEMS scanning units, which produces an image of 16 × 1 pixels (horizontal resolution) observed from one viewpoint. Reconstructed images for the MEMS scanning units are controlled using a computer and displayed via graphics boards. We evaluated the motion parallax of a 3D image by placing three light points at different positions in front of and behind the display plane. Spatial-formation 3D images of the points could be directly observed (see Method 2, Supplementary Figure 3, and Supplementary Video 1).

To highlight the LHE 3D display's capability, we extended the image by transforming the 1D arrays to 2D arrays with a multi-mirror array (see Method 3). A zigzag-shape-aligned multi-mirror array is used to reflect the projected light beams and realign extended 3D images in the 3D space (Fig. 3b). Thus, the display can show a 3D image with a horizontal resolution of 16 by N (N is the number of mirrors) pixels. Furthermore, since the image depth is too long (several meters in this study) to be displayed in a short distance, we use a reflection mirror to reflect the projected images and direct the light beams to a multi-mirror array.

**Figure 4:**
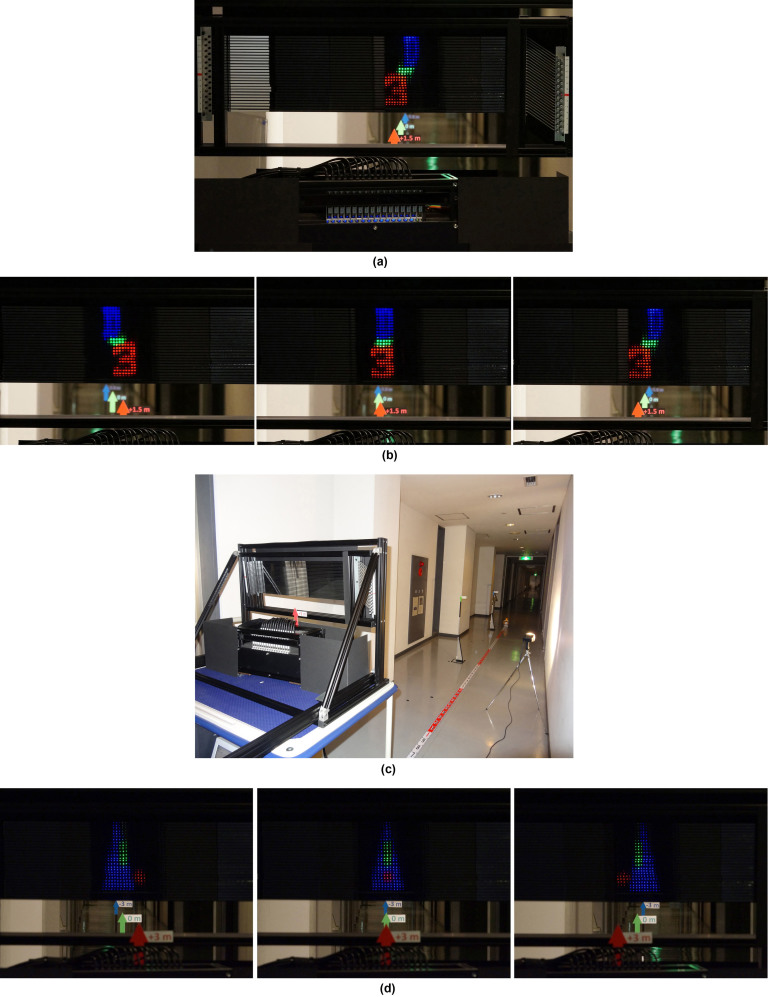
Evaluation of LHE 3D display's capability. (a) Manufactured MEMS scanning LHE 3D display device and displayed spatial formation 3D images with three markers at positions +1.5 m, 0 m, and −1.5 m. (b) Spatial formation 3D images viewed from left, center, and right, with camera zoom focused on position at +1.5 m. Moving seamlessly around display allows observers to see spatial formation 3D images from different perspectives with continuous motion parallax. (c) Experimental setup for evaluating image depths of LHE 3D display with three arrows: red, green, and blue at positions +3 m, 0 m and −3.0 m, respectively. (d) Motion parallax of long-viewing-distance LHE 3D images taken from various directions with camera zoom focused at −3.0 m (3 m inside screen); Red “circle”, 3 m from LHE 3D display; Green “|”, at position of LHE 3D display; Blue “▴”, 3 m inside LHE 3D display.

We evaluated the motion parallax of the 3D image by placing three letters at different positions in front of and behind the display plane. Figure 4a shows the prototype LHE 3D display and the displayed spatial formation 3D image. Images of “3”, “-”, and “D” located at the positions 1.5 m from the screen, on the screen, and 1.5 m inside the screen, respectively were produced. To compare the positions of the reconstructed images, we put three markers at +1.5, 0, and −1.5 m (symbol arrows showed in Fig. 4a, due to the reflection of the mirror and multi-mirror array, all arrows were placed behind the device. A detailed schematic of the experiment is available in Supplementary Figure 5). Figure 4b and Supplementary Video 2 show the motion parallax of the reconstructed LHE 3D images and those are fixed at the same position of the three markers.

Figure 4c shows another experiment to evaluate the possible depths of the displayed 3D image. Since the width of the prototype LHE 3D display is only 19 cm, the viewing area for observing a long-distance 3D image is small. In this study, we only presented a 3D image with six meters to ensure the viewing area, although the image depths can be enlarged. We placed three arrows: red arrow 3 m from the screen, green arrow at the screen, and blue arrow 3 m inside the screen (−3.0 m). The results showed that the LHE 3D display displayed a natural 3D image with super long image depth of six meters (Fig. 4d and Supplementary Video 3), which is a fantastic result compared with the original screen width of only about 19 cm.

Since we use a laser for light beam scanning, the LHE 3D display should theoretically have a longer viewing distance. The image depth and viewing area of the generated 3D image could be enlarged with more LHE MEMS scanning units and precision calibration of light rays. Furthermore, we can use galvano mirror scanning to enhance the flipping angle for the light beam; thus, create a larger viewing angle 3D display. Since such high-resolution images are replaced with electrical image signals from the MEMS scanning units, the 3D display can be produced using a relatively simple technology at a lower cost.

## Discussion and Summaries

Our LHE 3D display approach makes it easy to create a dynamic image at a video rate for potential future 3D telepresence. Because the LHE display approach does not require optical lenses or diffraction gratings that are commonly used for conventional 3D autostereoscopic displays, no optical aberrations are caused; therefore, it has the potential of creating a high spatial and temporal resolution 3D image. Of course, corresponding MEMS fabrication, mirror scanning, signal processing and display techniques should be developed to address the issue of separation of pixels. Furthermore, the LHE 3D display approach enables the production of a long-viewing-distance autostereoscopic image, which is an interesting and challenging area related to future 3D image display techniques.

Similar to the structure of digital micro-mirror devices, our MEMS scanning based LHE 3D display approach is possible to fabricate a flat-panel type 3D display with a large 3D image depth. Component units' integration technique, such as integrated array of light source, integrated colorization unit, array of MEMS scanning mirrors, can be used to make a compact and scalable MEMS unit array. By adjusting the MEMS scanning modes of the mirror and controlling the inputted signals accordingly, the LHE display approach is possible to generate 2D, binocular, multi-view, and integral 3D imaging with corresponding supplementary apparatus. The MEMS devices based 3D display can also be expected to solve accommondation-vergence conflict^20^. As well as to facilitate their entry into the field of general-purpose 3D imaging, LHE display technology may be used in medical image application^21^ and other information and communication fields.

We believe that this technology opens up the 3D display bottleneck for glasses-free autostereoscopic imaging, and make it possible to revolutionize the different forms of 3D naked-eye displays and 3D TVs. Furthermore, an LHE 3D display can be manufactured through the integration of MEMS and integrated circuit technology. Our LHE approach has the potential as an alternative to current 3D display technology that can produce multiple perspective views at full resolution.

## Methods

### Calibration of MEMS scanning array

We use a set of test patterns to calibrate the projected directions and positions of the MEMS scanning array. Supplementary Figure 1 shows the pattern (848 × 480 pixel) with a cross-shaped scale for calibration. Each MEMS scanning unit projects the test pattern onto a screen placed at different positions (Supplementary Figure 2). We use a camera to capture the images and then calculate the deviation of the projected images. The calibration information are used for both geometry correction and color balancing of the images projected from each MEMS scanning unit.

### Evaluation of LHE 3D display

We evaluated LHE 3D display by directly observed spatial formation 3D images (points). Supplementary Figure 3a shows the prototype LHE 3D display and displayed spatial formation 3D images (points). The spatial formation of three points: Red, Green, and Blue located 3 m from the screen, 1.5 m from the screen, and 1.5 m inside the screen, respectively. To compare the positions of the reconstructed images, we put markers at +3, and +1.5 m (the arrows shown in Supplementary Figure 3b). Since blue point is inside the screen, we did not place blue arrow (−1.5 m) in experiment. Supplementary Figures 3c and Supplementary Video 1 show that the motion parallax of the reconstructed images (points) is fixed at the same position of the markers.

### Multi-mirror array for transforming one-line MEMS scanning array into multi-line MEMS scanning array

We developed a prototype LHE 3D display by aligning a set of MEMS scanning units in the horizontal. Our first experiment (as shown in Method 2 and Supplementary Figure 3) demonstrated the special formation of an LHE 3D image with only one-line horizontal information. We extended the image to the vertical direction. The images are projected from the MEMS scanning unit with full information from different viewing directions. The MEMS unit is generating multiple views in its horizontal scan and multiple scanlines in its vertical scan. The light beam in the unused vertical direction is reflected on a multi-mirror array, so that the image can be extended to the vertical direction and be observed. We confirm that the 3D display can spread up and down left and right. Supplementary Figure 4 shows a conceptual configuration of using a multi-mirror array to generate a vertically extended MEMS scanning array. The MEMS scanning unit array is aligned horizontally. A multi-mirror array with a small height of 5 mm and a width of 400 mm is used to extend the vertical direction of the MEMS scanning array. By arranging N (N = 27 in this study) sheets of flat mirror in an arc, the one-line MEMS scanning units can be observed as a stage N stacked vertical unit. Moreover, because the different positions in the vertical are viewed from the MEMS scanning unit array, we can observe the pixels of different heights in the image of each MEMS scanning unit. Based on this configuration, it is possible to collect perspective rays emitted in different directions vertically from the MEMS scanning unit array. It looks as if the mirror images from the horizontally aligned MEMS scanning unit array are stacked up and down as a set of vertically extended MEMS scanning arrays. This is similar to a 2D arrangement of a MEMS scanning unit array. The experimental setup for evaluation of motion parallax and long-viewing-distance of the LHE 3D images are also shown in Supplementary Figure 5.

## Supplementary Material

Supplementary Information Supplementary Information

Supplementary Videos Supplementary Video 1

Supplementary Videos Supplementary Video 2

Supplementary Videos Supplementary Video 3

Supplementary Videos Supplementary Video 4

## Figures and Tables

**Figure 1:**
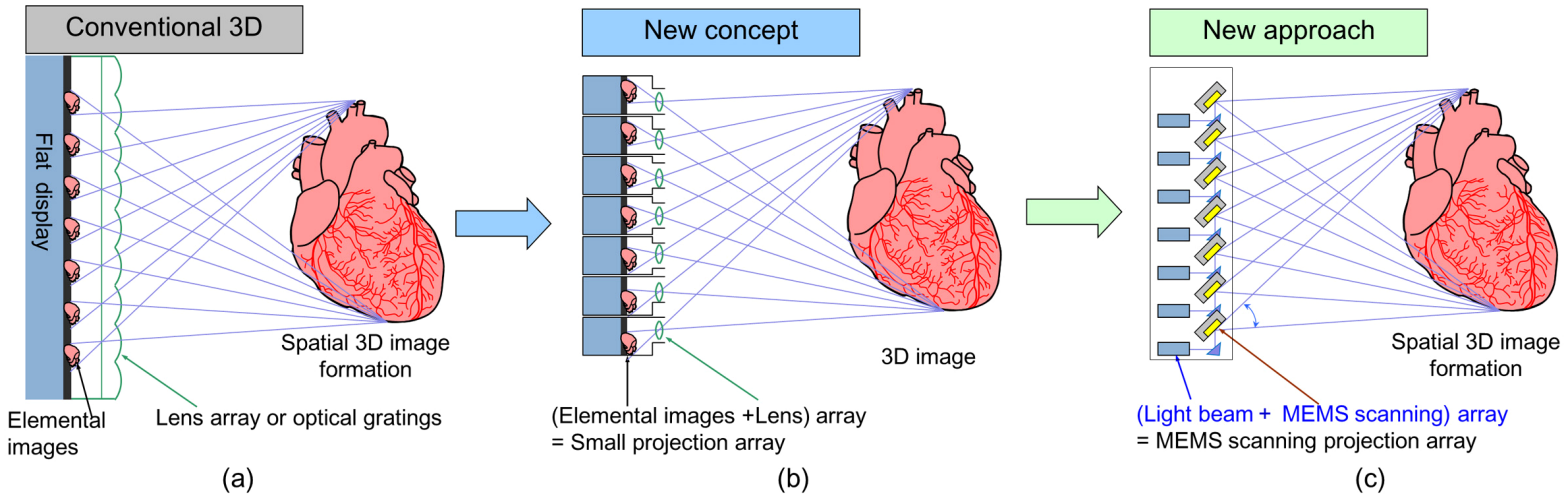
Proposed approach using light homogeneous emitting (LHE) with MEMS scanning array instead of lens array and flat-panel display to generate 3D images. (a) Conventional 3D displays uses elemental images and lens array or grating for 3D images generation. (b) 3D imaging can be interpreted as a method for displaying 3D objects at a desired spatial position by emitting light rays through a small projection array comprising an elemental image array and projecting lens array. (c) With proposed 3D display approach, MEMS scanning units are formed in the same size as that of the small projection array arranged in (b). Light beams are always scanned two-dimensionally and repeatedly but intermittently transmitted to the viewing area.
