# Supplementary material for: Super long viewing distance light homogeneous emitting three-dimensional display
Source: Sci Rep. 2015 Apr 1;5:9532. doi: 10.1038/srep09532 (PMC5383051; doi:10.1038/srep09532)
Supplement: Supplementary Information [file srep09532-s1.pdf]

Supplementary Information

Super long viewing distance light homogeneous emitting three-dimensional display

Hongen Liao*

Department of Biomedical Engineering, School of Medicine, Tsinghua University, Beijing 100084, China

Corresponding author: H. L. (liao@tsinghua.edu.cn)

Supplementary Figures

Supplementary Figure 1

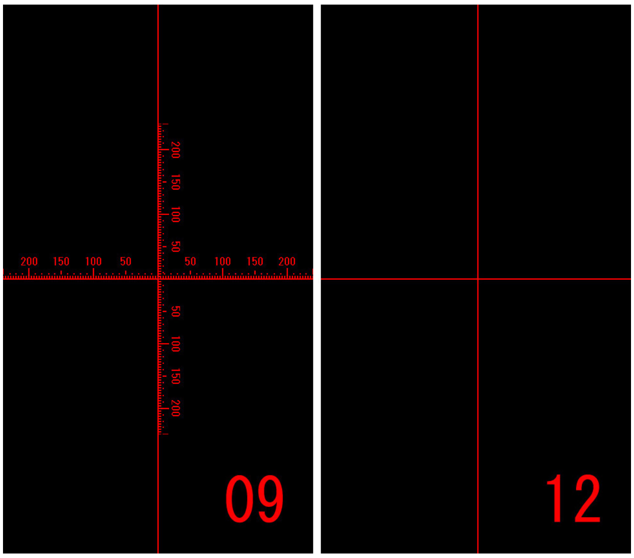

Supplementary Figure 1 | Patterns for MEMS scanning unit calibration. Patterns for MEMS scanning unit projection image calibration, figures only show the patterns for unit No. 9 (with a cross-shaped scale) and unit No. 12 (without scale).

Supplementary Figure 2

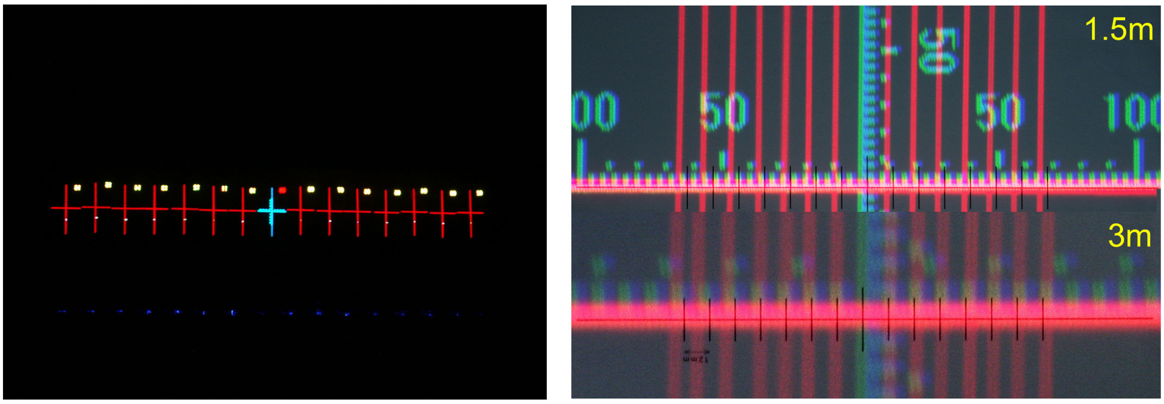

(a) (b)

Supplementary Figure 2 | Test pattern images projected from each MEMS scanning unit. Deviation of the projected images are calibrated by a camera. Each MEMS scanning unit projects the test pattern onto a screen placed at different positions. (a) Screen is placed near MEMS scanning array. (b) Screen is placed at 1.5 m and 3 m from MEMS scanning array. Figure shows the overlaid images projected from MEMS scanning units after geometry correction and color balancing .

Supplementary Figure 3

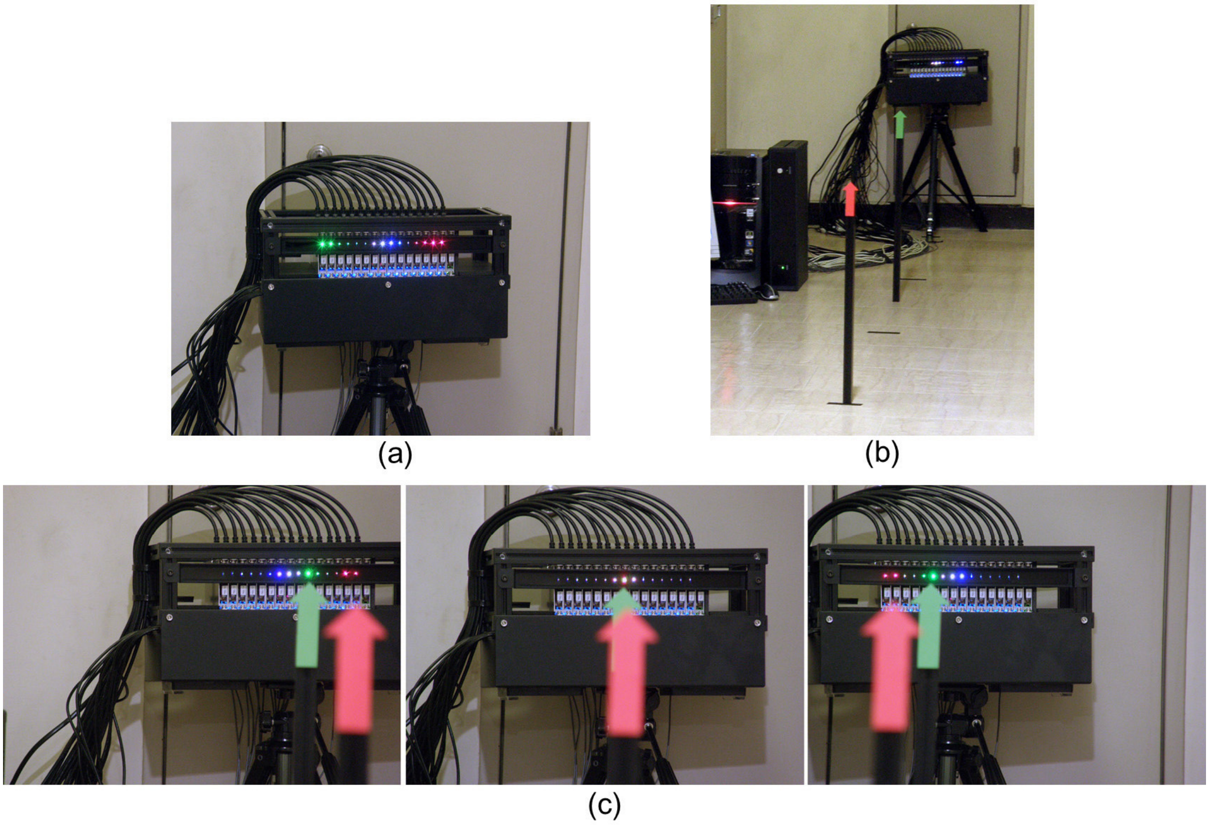

Supplementary Figure 3 | Evaluation of LHE 3D display by directly observed spatial formation 3D images. (a) Prototype LHE 3D display and displayed spatial formation 3D images with three points at different positions. (b) Experimental setup for evaluating image depths provided by LHE 3D display, with two reference arrows: red arrow 3 m from screen and green arrow 1.5 m from the screen. (c) Evaluation experimental results of image depths generated using the LHE 3D display. Image position of red point is at same position of red arrow; 3 m from screen, green point is at same position of green arrow; 1.5 m from screen, and blue point is at 1.5 m inside screen. Motion parallax of the special formation images (points) can be directly observed by naked eye.

Supplementary Figure 4

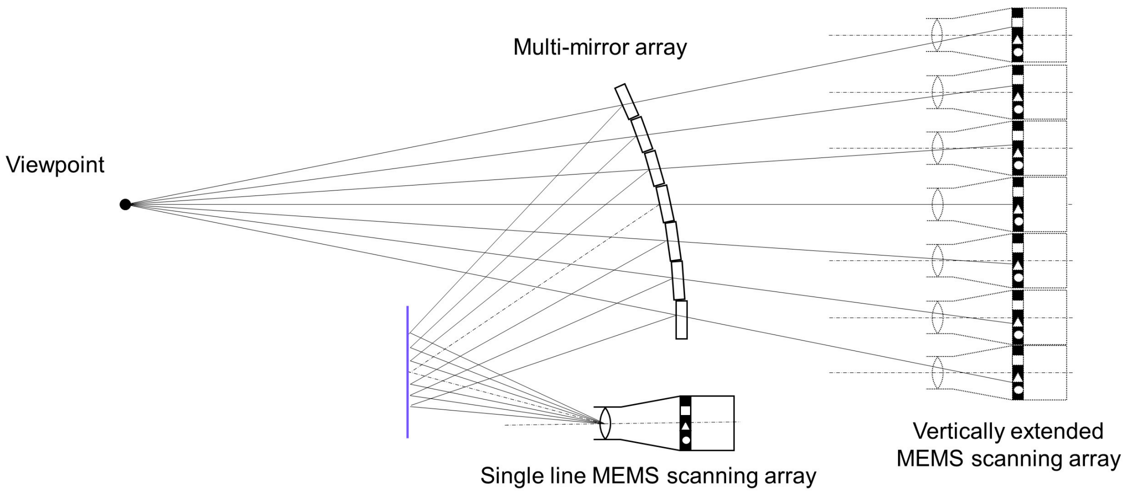

Supplementary Figure 4 | Multi-mirror array for generation of vertically extended MEMS scanning array. 3D display conceptual configuration that combines multi-mirror array and MEMS scanning unit array to generate vertically extended MEMS scanning array. Multi-mirror array designed for increasing vertical dimension of extension images. 3D display conceptual configuration that combines a mirror array and MEMS scanning unit array.

Supplementary Figure 5

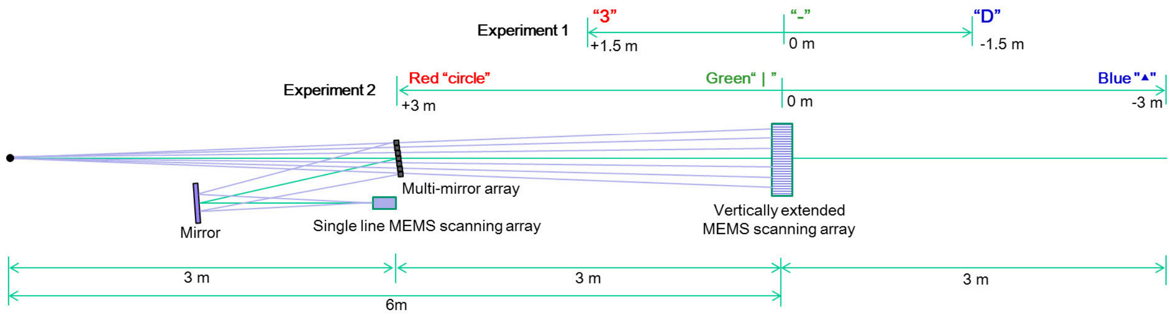

Supplementary Figure 5 | Experimental setup for evaluation of LHE 3D images. Experiment 1 for evaluation of motion parallax of the 3D images (as shown in Figure 4a): Images of “3”, “-”, and “D” located at the positions 1.5 m from the display, on the screen, and 1.5 m inside the screen, respectively. Experiment 2 for evaluation of the long viewing distance of LHE 3D images (as shown in Figure 4c): Images of Red "circle", Green" | ", and Blue "▲" located at the positions 3 m from the display, at position of display, 3 m inside the display, respectively.

Supplementary Videos

Supplementary Video 1. This video shows the reproduced LHE 3D images (points). Motion parallax of the special formation images (points) can be directly observed by naked eye, as displayed in Supplementary Figure 2.

Supplementary Video 2. This video shows motion parallax of displayed 3D images, position relationship between three markers at +1.5 m, 0 m, and -1.5 m, and displayed 3D images are not changed even if we move the camera, as displayed in Figure 4b of manuscript.

Supplementary Video 3. Animated movie of displayed 3D images, position relationship between three markers at +1.5 m, 0 m, and -1.5 m, and displayed 3D images are not changed even if we move the camera.

Supplementary Video 4. This video shows motion parallax of reproduced LHE 3D images and demonstrates that the LHE 3D display displayed a natural 3D image with super long image depth of six meters, as displayed in Figure 4d of manuscript.
